# Fostering shared decision making by occupational therapists and workers involved in accidents resulting in persistent musculoskeletal disorders: A study protocol

**DOI:** 10.1186/1748-5908-6-22

**Published:** 2011-03-17

**Authors:** Marie-France Coutu, France Légaré, Marie-José Durand, Marc Corbière, Dawn Stacey, Patrick Loisel, Lesley Bainbridge

**Affiliations:** 1 Centre for action in work disability prevention and rehabilitation (CAPRIT) and School of Rehabilitation, Université de Sherbrooke, 1111, rue St-Charles ouest, bureau 101, Longueuil (Québec), J4K 5G4C, Canada; 2 Research Center of Centre Hospitalier Universitaire de Québec, Hospital St-François d'Assise, CHUQ, 10 rue Espinay Québec (Québec), G1L 3L5, Canada; 3 Department of Family Medicine and Emergency Medicine, Faculty of medicine, Université Laval, Pavillon Landry, avenue de la medicine, Québec (Québec), G1K 7P4, Canada; 4 School of Nursing, Faculty of Health Sciences, University of Ottawa, Guindon Hall, 451 Smyth Road, Ottawa, ON, K1H 8M5, Canada; 5 Dalla Lana School of Public Health, University of Toronto 155 College Sreet, 5th Floor, Toronto (Ontario), M5T 3M7, Canada; 6 Faculty of Medicine, College of Health Disciplines 400 - 2194 Health Sciences Mall, Vancouver (British Colombia), V6T 1Z3, Canada

## Abstract

**Background:**

From many empirical and theoretical points of view, the implementation of shared decision making (SDM) in work rehabilitation for pain due to a musculoskeletal disorder (MSD) is justified but typically the SDM model applies to a one on one encounter between a healthcare provider and a patient and not to an interdisciplinary team.

**Objectives:**

To adapt and implement an SDM program adapted to the realities of work rehabilitation for pain associated with a MSD. More specific objectives are to adapt an SDM program applicable to existing rehabilitation programs, and to evaluate the extent of implementation of the SDM program in four rehabilitation centres.

**Method:**

For objective one, we will use a mixed perspective combining a theory-based development program/intervention and a user-based perspective. The users are the occupational therapists (OTs) and clinical coordinators. The strategies for developing an SDM program will include consulting the scientific literature and group consensus with clinicians-experts. A sample of convenience of eight OTs, four clinical coordinators and four psychologists all of whom have been working full-time in MSD rehabilitation for more than two years will be recruited from four collaborating rehabilitation centres. For objective two, using the same criteria as for objective one, we will first train eight OTs in SDM. Second, using a descriptive design, the extent to which the SDM program has been implemented will be assessed through observations of the SDM process. The observation data will be triangulated with the dyadic working alliance questionnaire, and findings from a final individual interview with each OT. A total of five patients per trained OT will be recruited, for a total of 40 patients. Patients will be eligible if they have a work-related disability for more than 12 weeks due to musculoskeletal pain and plan to start their work rehabilitation programs.

**Discussion:**

This study will be the first evaluation of the program and it is expected that improvements will be made prior to a broader-scale implementation. The ultimate aim is to improve the quality of decision making, patients' quality of life, and reduce the duration of their work-related disability by improving the services offered during the rehabilitation process.

## Background

For individuals having a persistent work disability due pain associated with a musculoskeletal disorder (MSD), a return to work (RTW) will depends on the complex interaction among several types of factors: biological (*e.g.*, medical status, physical capacities), psychological (*e.g.*, fears, beliefs, self-efficacy), and social (*e.g.*, work environment, interaction among rehabilitation professionals, and management and compensation policies) [1,2]. Therefore, interventions will have to focus primarily on the reduction of the work disability rather than pain reduction. However, a qualitative study observed that when referred to an interdisciplinary work rehabilitation program injured workers expected complete pain alleviation [3]. In the absence of agreement, the clinician and patient were not focused on the same action plan and did not use the same criteria for evaluating treatment efficacy [3,4]. Consequently, this paradigm change has important implications for clinical practice and for the establishment of an alliance with the patient/injured worker because the gap between workers expectancies and what is being offered has evidence-based treatment can be significant.

### Shared decision making to prevent clinician-patient gaps

Through shared decision making (SDM), it may be possible to reduce gaps such as in understandings/representations, values, and expectancies between clinicians and patients. SDM is currently defined by the joint process of decision making of patient and clinician, in which information is exchanged, preferences are expressed and discussed, and agreement is reached regarding the goals and action plan to pursue. Follow-up is also planned for the purpose of evaluating and, if necessary, readjusting the goal or the plan in place [5]. A systematic review of the barriers and facilitators of implementing SDM, with data from 39 studies in 15 countries, did not reveal a single study in rehabilitation, thus underscoring a major knowledge gap [6]. This review also brought to light the three main barriers to implementing SDM, namely, time constraints and problems in applying the process due to the patients' characteristics or to those of the clinical setting [6]. These findings therefore highlight the importance of including the practice settings in the different steps involved in implementing an SDM process.

Through the exchange of information and discussion of preferences, the SDM process seeks to improve both patient's and clinician's decisional conflict, thus improving the quality of the decision itself and reducing uncertainty when the clinician cannot guarantee a specific result [7]. The level of decisional conflict is defined by the uncertainty associated with an action, in cases where a choice must be made among different options (*e.g.*, options for returning to work) that may involve a risk, loss, or regret, or go against personal values. The Ottawa Decision Support Framework (ODSF) [7] specifically addresses the decisional conflict [7]. This model was developed to improve the quality of decisions made in the health context by addressing the determinants of decisions and endeavouring to act on the modifiable factors. The determinants of decision-making conflict comprise the patient's and clinician's characteristics, as well as perceptions of the decision that has to be made, social pressure/support to make a decision, and resources needed to implement the decision [7]. These determining factors were also observed in prior studies on patients with work disabilities [3,8]. These findings therefore underscore the need to aid workers having a work disability in order to enhance the quality of the decisions made by reducing uncertainty and improving the decision-making process. In fact, several studies have noted a significant correlation between reducing patients' decisional conflict and an improved understanding of their problem, as well as a reduction in regrets and blame of the clinicians [9].

### Conceptual framework

**Figure 1:**
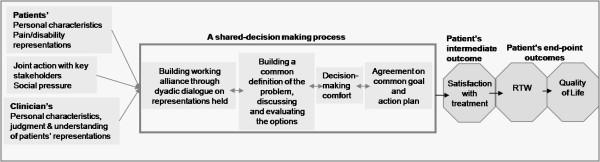
**The project's conceptual framework seen from a patient perspective**.

Based on findings in disability prevention studies, in empirical data in health psychology and psychotherapy, and SDM literature, we therefore propose the general conceptual framework depicted in Figure 1 as the basis of this study protocol.

The objective of this project is to adapt and implement an SDM program adapted to the realities of work rehabilitation for persistent pain associated with an MSD. This will allow for a first evaluation of the program so that improvements can be made prior to a broader-scale implementation during a second phase. The ultimate aim is to improve the quality of decision making, patients' quality of life, and reduce the duration of their work-related disability by improving the services offered during the rehabilitation process. To facilitate attainment of the project objective, we will pursue the following two specific objectives: to adapt an SDM program applicable to existing work rehabilitation programs; and to evaluate the extent of implementation of the SDM program in four work rehabilitation clinics.

## Methods

### General methodology

This study falls into the field of evaluative research [10]. Program development requires four iterative phases: needs evaluation; program planning and development; program implementation and evaluation; and program improvement [11]. Each of these cycles involves a specific methodology and specific analyses to ensure the validity and reliability of the data collected [12]. In the first cycle, needs were evaluated using a deductive approach based on our prior studies [3,8]. The current project therefore begins in the second cycle, that of program planning and development.

### Specific objective one: To adapt an SDM program applicable to existing work rehabilitation programs

#### Design

A mixed perspective, which we have used successfully in prior studies, will combine a theory-based perspective [13] and a user-based perspective [14]. In this project, the users are occupational therapists (OTs) and clinical program coordinators. The strategies used to adapt the SDM program/intervention will be defined using the scientific literature and a group-consensus method [15].

By adopting a mixed perspective, combining those of rehabilitation theory and users, we will be able to adapt an SDM program/intervention adapted to the realities of rehabilitation practice. Over and above the users we have identified (OTs and clinical program coordinators), we believe that the addition of psychologists, with their expertise regarding the establishment of a working alliance, will significantly contribute to the adaptation of the SDM program.

#### Participants

A convenience sample comprised of eight OTs, four coordinators, and four psychologists will be recruited from the following four large rehabilitation centers that have agreed to collaborate: the Centre de réadaptation de l'Estrie; Centre Montérégien de Réadaptation; Centre de réadaptation Lucie Bruneau; and the Centre Hospitalier de l'Université de Sherbrooke. These centres were chosen because they all apply the same work disability evidence-based intervention principles [16]. They will also provide a variety of expertise, taking into account the culture and the context of different referring agencies, such as the Quebec's workers compensation board, the Québec automobile insurance agency, the public health insurance, and the private insurers. The number of experts retained is representative of their distribution in the practice settings. The inclusion criteria are as follows: working full-time in MSD rehabilitation for more than two years as psychologists, OTs, or clinical program coordinators. These inclusion criteria were based on our prior studies [14], as in the past they have permitted recruitment feasibility while providing substantial information. Also, the number of participant will help achieve data saturation, while maintaining a good dynamic in the group [17].

#### Data collection and analysis

For the theory-based perspective, a first conceptual framework for the SDM process was developed based on a literature review and our empirical data. Using this conceptual framework, a first theoretical version will be operationalized [13] to define the specific objectives of the SDM process. We will then be in a position to draw up a detailed plan [13] relating the objectives to the activities and resources needed for the SDM program.

Using this theoretical version, the users will be consulted to allow us to adapt the theory to the realities of work rehabilitation. For this purpose, the Technique for Research of Information by Animation of a Group of Experts (TRIAGE) method [15] will be used. This group consensus method allows data to be studied, compiled, and analyzed by stimulating reflection among experts. First, the experts will individually study the program theory that has been derived from the literature and will complete a questionnaire on the theory. The questionnaire will seek first to document their level of agreement regarding some of the statements about the program. Rated on a 4-point scale (totally disagree to totally agree) [18]. When respondents gives a rating of less than 3, which corresponds to disagree and totally disagree, they will be asked to make a maximum of five suggestions of ways to improve the program. For example, here are the statements and specific questions that could be asked [13,19]:

1. The objectives are necessary and essential for a share decision-making process in work rehabilitation. Propose objectives or clarifications of the objectives presented that you deem essential for a SDM process in work rehabilitation; Identify the objectives that are non-essential and that should be removed.

2. The objectives are realistic for a context of SDM in work rehabilitation. Please make the necessary changes to the objectives that are not realistic.

3. The activities are essential, related to the objectives, and both realistic and feasible. They are formulated in clear and concrete terms. Propose activities or clarifications of the activities presented that are related to the objectives, realistic, and feasible, formulating them in clear and concrete terms; Identify activities that are non-essential and that should be removed.

4. The indicators proposed are necessary, sufficient, and appropriate to measure the attainment of the objectives. Propose essential indicators or clarifications of the essential indicators presented that are related to the objectives; Identify the indicators that are non-essential and should be removed.

5. The resources (human and material) proposed will make it possible to attain the objectives and carry out the activities. They are also sufficient. Propose resources that are essential to attaining the objectives and carrying out the activities.

6. Regarding the implementation of the SDM program, the following questions will be asked [20]: The program fits in with current practices; What factors will hinder implementation? What factors will facilitate implementation?

The participants' answers will be compiled in preparation for the group meetings. These answers will be written on separate cards, and be discussed in a series of approximately four meetings of the same participants. Each meeting will be limited to a maximum of three hours. At the outset, the participants will be told not to try to produce a perfect SDM program. The second specific objective (first implementation) will enable certain components of the SDM program to be further clarified or possibly modified.

The meetings will be recorded to support the notes taken by the research assistant on the group's decisions. A summary will be written up for each group meeting and the literature may be consulted in order to document the emerging data. After the group meetings have been completed, an SDM program adapted to work rehabilitation will be ready for a first implementation.

The study protocol has been approved by all affiliated research ethics committees of the participating rehabilitation centres. All experts who agreed to participate to the group consensus will sign an informed consent form. The group consensus session will be held during working hours. The research project will therefore provide financial compensation for the loss of clinical activity time.

### Specific Objective two: Evaluate the extent of implementation of the SDM program in four work rehabilitation clinics

#### Design

In order to evaluate the extent of implementation, an exploratory study with a descriptive design will be used [21]. The extent of implementation corresponds to the gap between what is planned and what is offered in reality [22]. In order to implement the program within the rehabilitation programs at the four centres, the OTs will be offered training on SDM. This training component will help standardize the SDM process. The extent of implementation will be evaluated on the basis of the audiotaped observation of the SDM process carried out by the OT/patient dyad. The results of the observations will be triangulated using both the results of a self-administered questionnaire completed by the members of the dyad and an individual interview with each OT after he or she has completed follow-up of five patients. The design will make it possible to identify the reasons for the gap between what is prescribed and what is offered. The program and training offered can then be modified accordingly.

#### Participants

A total of eight OTs will be recruited and trained according to the criteria associated with specific objective one. OTs were selected as the relevant professionals because they conduct the initial diagnostic evaluation when a patient is referred to the rehabilitation centre. Also, they are frequently the main health professional involved in the rehabilitation process. The OTs will not be obliged to have participated in the specific objective one phase, given that training will be offered. Moreover, since the OTs have already displayed their interest in participating in this study, according to the innovation dissemination model, they are considered to be 'early adopters,' thus facilitating implementation [23].

Five patients will be recruited for each OT, for a total of 40 patients. The patients will have to be of working age (between the ages of 18 and 64) have been off work for more than 12 weeks due to pain associated with a MSD, and be starting a work rehabilitation program. To promote external validity, only patients with a specific MSD (recent fracture, metabolic disease, neoplasia, inflammation, or infection of the spinal column) will be excluded.

#### Training the OTs in the SDM process

SDM training derived from theories on innovative intervention implementation [6,24] will be adapted to the rehabilitation context. This training will be given in an interactive one and a one-half hour workshop that has been successfully implemented in Quebec [25]. A before and after study of the impact of this training has also observed an improvement in physician/patient agreement [24]. According to the basic principles of the Ottawa Decision Support Framework [7], the objectives of training are as follows: identify the modifiable determinants contributing to decisional conflict; provide decision support to patient needs; and learn to use the validated generic decision-aid instrument based on the Ottawa Framework [26]. In addition to this training on basic SDM skills [27,28], advanced training on the working alliance will be offered based on graduate course taught by the principal investigator and offered online with a one-day synthesis of the learning done in class. The course was developed with the help of a techno-pedagogue.

#### The rehabilitation program

The SDM program will be implemented in the four participating centres that currently offer similar evidence-based rehabilitation programs. In work disability prevention, evidence-based intervention principles include staying physically active [29], reassuring patients about their MSD [30,31], reducing both fears and avoidance of pain and movement [32,33], implementing a progressive RTW [16,34,35] and collaboration with stakeholders [36,37]. The main steps in rehabilitation programs are the initial diagnostic evaluation, the clinical phase, and the therapeutic RTW, which includes gradual *in vivo *exposure. The specific moment during the rehabilitation program for offering the SDM will be clarified during the specific objective one phase. However, it is realistic to think that it will be done at the beginning of the rehabilitation program, following the initial evaluation but prior to establishing the treatment plan. As the patients are referred by a third party (private or public insurers), the referring party expects the goal of the rehabilitation to be a RTW. This goal cannot therefore be modified. In fact, patients who refuse to RTW may lose entitlement to their income replacement indemnities. Despite this situation, our prior studies show that other important decisions can be made [8,38]. The SDM process will then focus on the options pertaining to a healthy, safe, and sustainable RTW.

#### The recruitment procedure

The clinical program coordinators at the four centres will be our key informants, helping us to identify potential participants in light of the patient inclusion and exclusion criteria. Two weeks prior to the initial diagnostic evaluation, if an OT trained in SDM is asked to evaluate an eligible participant, the coordinator will request the patient's consent to being contacted by a research assistant who will describe the research project. The research assistant will then contact the person by telephone to present the details of the study. Should the patient be interested in participating, the research assistant will meet with the patient before the initial evaluation to have the consent form signed and to answer any questions the participant may have. This procedure will enable the patient to make a clear distinction between the research process and the usual clinical interventions.

#### Data collection procedure

The patients who agree to participate will undergo their initial diagnostic evaluation as planned in the clinical procedures. Toward the end of this meeting, the SDM process will be launched. This last segment of the meeting will be audiotaped by the OT to avoid bringing a third person into the meeting and possibly changing the dyadic dynamic. At the end of the meeting, the research assistant will take the recording to be transcribed and will invite the participant and OT to complete a self-administered questionnaire independently and confidentially.

Once an OT has completed this procedure with five participants, he or she will be asked to participate in a semi-structured individual interview for the purpose of documenting the factors hindering and facilitating implementation of the SDM process. The same research assistant will conduct the interviews.

Informed consent will be sought from the injured workers. The OTs will have to sign a new consent form each time a patient agrees to be involved in the SDM process. The training in SDM will be held during working hours. The research project will therefore provide financial compensation for the loss of clinical activity time.

#### Variables and measurements

##### SDM-related activities

The SDM activities carried out by the OT/patient dyad will be recorded and evaluated using the OPTION observation scale [39], for which a validated French language translation already exists [40]. Using this scale and audiotaped recordings of the SDM, it is possible to rate the 12 basic skills needed for SDM on a five-point scale from 0 (behaviour not observed) to 4 (skill observed and exhibited to a high standard). The scale has good construct validity and [41] and has yielded very high inter-rater agreement, with intra-class correlation coefficient scores of 0.77 [39]. High internal consistency has also been observed [41]. A systematic review of SDM observation instruments shows this scale to have the best psychometric qualities [42].

##### Working alliance

To triangulate the observational data, a validated dyadic questionnaire on the working alliance [43] will be filled out by each member of the dyad. It will allow the evaluation of each person's perception of the quality of the relationship established. The questionnaire has 12 items rated on a Likert scale and measuring the perception of the relationship established, of the task performed, and of the goals pursued. High Cronbach's alphas have been observed [43] for each of the three constructs, and ranged from 0.83 to 0.98.

##### Barriers to and facilitators of implementation of SDM

Semi-structured individual interviews will serve to document the OTs' perception of the factors hindering and facilitating SDM implementation. The following questions will be asked: Are there typical cases in which the SDM process worked particularly well? What were the characteristics of these cases (*e.g.*, patient-related, legal or administrative context, *et al.*)? Are there typical cases in which the SDM process did not work particularly well? What were the characteristics of these cases (*e.g.*, patient-related, legal or administrative context, *et al.*)? Which prescribed objectives or activities in the SDM program theory did you feel most comfortable using? With which ones did you feel least comfortable? Why? Can you make any suggestions to improve the program theory?

At the end of these questions, the interviewer will present the interviewee with the aggregate results of the OPTION scale ratings for the five cases he or she is managing, to maintain the confidentiality of the individual results. These results will be presented in a respectful manner to avoid blame and to promote the emergence of explanations and theories as to the differences between what was done and what was prescribed.

#### Data analysis

Descriptive analysis will be performed on the scores of the questionnaire completed by each member of the dyads. The transcript of the 40 SDM processes will be analyzed by two independent evaluators using the OPTION rating scale to obtain a score for implementation. Qualitative data analysis of the individual interviews with the OTs will be done to determine whether the OTs carried out the SDM process successfully as prescribed in the program theory. For this purpose, content analysis will be performed using a list of *a priori *codes. These codes will come from a taxonomy of barriers and facilitators of implementation of SDM [6]. However, the emergence of new codes in light of the data obtained will be possible, in an effort to remain as faithful as possible to the transcript. Qualitative data analyses will be done using ATLAS-TI analysis software [44]. The interviews will be coded by a research assistant and the principal investigator to obtain inter-rater agreement using the Landry method [45]. The codes assigned to the excerpts will then be compared and divergences discussed in order to clarify any ambiguities. This process will be carried out until inter-rater agreement of 90% [46] is obtained. A summary will be prepared of the barriers and facilitators identified by each OT, and the summaries then compared to identify points of convergence and divergence between cases. Following this step, discussion groups will be held with all the researchers and the research assistant for the purpose of reaching a consensus on the extent of implementation, and on the barriers to and facilitators of implementation. Depending on the findings, the program theory will be improved and/or additional training given.

## Discussion

Current studies across the various health fields in SDM only offer objectives or general recommendations [47]. To the best of our knowledge, no SDM program exists that presents an operationalized conceptual framework relating objectives to specific activities and resources. The findings of our project will make it possible to fill this gap in the literature.

This is a pragmatic study involving a limited number of clinicians. Therefore, further generalization and confirmation of the findings will be necessary with additional clinical environments and larger subject groups. This study, however, is a necessary step because the content of the innovative SDM process must be adapted to work in the disability prevention field. We realize that the conceptual framework is a simplified model addressing only a portion of the complex interactions between the stakeholders and patients in the work disability process.

The design and multiple theory-driven basis of this study will help prevent the problem that frequently arises during the evaluation of complex interventions: that the program was not clearly defined or not thoroughly developed [48]. We will also have gathered information from three sources (observation, patients, and OTs) to increase the reliability of the evaluation of the extent of implementation. We have previously validated and used these triangulation measures [3,40,43,49]. Combining theory-driven and user-based perspectives will also reduce the main barriers to SDM implementation: lack of applicability due to the clinical situation, and lack of applicability due to patient characteristics [6]. Therefore, in-depth documentation of the implementation process with a view to improving the program will contribute to the success of a future, broader-scale implementation during a second phase. In addition, this project will facilitate implementation of the shared decision-making program in the context of other problems generating work disability, such as mental health problems.

## Competing interests

The authors declare that they have no competing interests.

## Authors' contributions

MFC wrote the study protocol and conceived the study. FL contributed with her expertise and in the writing on SDM concept and training, as well as identifying the assessment of the SDM activities. MJD brought her expertise in the methods section on program evaluation, she contributed to the elaboration of the interdisciplinary work rehabilitation program and acted has a mentor to MFC. MC participated with his expertise in working alliance concept and its assessment. DS contributed with her expertise on SDM concepts and training. PL participated with his expertise on work disability prevention he contributed to the elaboration of the interdisciplinary work rehabilitation program and acted has a mentor to MFC. LB brought her expertise in interprofessional education and knowledge transfer expertise in clinical settings. MFC is its guarantor. All authors contributed in obtaining the funding. All authors read, edited, and approved the final manuscript
